# Patterns and evolution of ACGT repeat *cis*-element landscape across four plant genomes

**DOI:** 10.1186/1471-2164-14-203

**Published:** 2013-03-25

**Authors:** Rajesh Mehrotra, Sachin Sethi, Ipshita Zutshi, Purva Bhalothia, Sandhya Mehrotra

**Affiliations:** 1 Biological Sciences Department, Birla Institute of Technology and Science, Pilani, RJ, India

**Keywords:** Gene expression, Promoter regulation, *Cis*-element, Stress, Spacer, *Arabidopsis*, Rice, Soybean, Sorghum, Inter-motif distance

## Abstract

**Background:**

Transcription factor binding is regulated by several interactions, primarily involving *cis*-element binding. These binding sites maintain specificity by means of their sequence, and other additional factors such as inter-motif distance and spacer specificity. The ACGT core sequence has been established as a functionally important *cis*-element which frequently regulates gene expression in synergy with other *cis*-elements. In this study, we used two monocotyledonous – *Oryza sativa* and *Sorghum bicolor*, and two dicotyledonous species – *Arabidopsis thaliana* and *Glycine max* to analyze the conservation of co-occurring ACGT core elements in plant promoters with respect to spacer distance between them. Using data generated from *Arabidopsis thaliana* and *Oryza sativa,* we also identified conserved regions across all spacers and possible conditions regulating gene promoters with multiple ACGT *cis*-elements.

**Results:**

Our data indicated specific predominant spacer lengths between co-occurring ACGT elements, but these lengths were not universally conserved across all species under analysis. However, the frequency distribution indicated local regions of high correlation among monocots and dicots. Sequence specificity data clearly revealed a preference for G at the first and C at the terminal position of a spacer sequence, suggesting that the G-box motif is the most prevalent for the ACGT class of promoters. Using gene expression databases, we also observed trends suggesting that co-occurring ACGT elements are responsible for gene regulation in response to exogenous stress. Conservation in patterns of ACGT (N) ACGT among orthologous genes also indicated the possibility that emergence of functional significance across species was a result of parallel evolution of these *cis*-elements.

**Conclusions:**

Although the importance of ACGT elements has been acknowledged for several plant species, ours is the first study that attempts to compare their occurrence across four species and analyze conservation among them. The apparent preference for particular spacer distances suggest that these motifs might be implicated in important physiological functions which are yet to be identified. Variations in correlation patterns among monocots and dicots might arise out of differences in transcriptional regulation in the two classes. In accordance with literature, we established the involvement of co-occurring ACGT elements in stress responses and showed how this regulation differs with variation in the ACGT (N) ACGT motif. We believe that our study will be an essential resource in determining optimum spacer length and spacer sequence between ACGT elements for promoter design in future.

## Background

Eukaryotic gene expression and its regulation by means of transcription is one of the most significant areas of research currently. Regulation of gene transcription relies on interactions among transcription factors (TFs) which bind to specific DNA *cis* sites to form a conglomeration of proteins which guide the polymerase binding [1]. These *cis* sites include enhancers, core promoters, matrix or scaffold attachment regions, insulators and silencers. Of these, enhancer elements are an important class of *cis-*regulatory sequences that are usually present upstream of the transcription initiation site and contain multiple short binding site sequences for targeting several activators and repressors of transcription. These short binding site sequences are often referred to as sequence motifs and occur in recurring patterns across DNA [2]. It is generally accepted that sequence motifs co-evolve with their core promoter in both sequence specific and location specific ways to achieve a directed target function [3,4]. Comparative studies show conserved regions of promoters are expressed widely across genomes of various species, indicating directed evolution [5], correlating with the notion that functionally less important regions of DNA evolve (in terms of mutant substitutions) faster than more important ones [6]. For example, in a comparison of the 200 base pair early enhancer of *Hoxc8* in 29 species of mammals the complete nucleotide sequences of this region were 90% similar across all taxa, confirming that this enhancer sequence has been specifically conserved [7,8]. Apart from the sequence of these motifs, the positional and inter-motif distances within the enhancers also play a critical role in interactions between transcription factors [9], as the spacing and intermediary sequences control the size and strength of interactions of TF binding [10], subsequently affecting the gene expression [11]. In fact, unless helical phasing is conserved to provide allowance for protein binding, even a change in spacer length by one base pair can drastically alter gene expression levels [12]. Holistically, this implies that conserved sequences (conforming to the various spatial and positional constraints) occurring in higher numbers as compared to what is probabilistically expected may be of specific functional significance.

In plant genomes, one such sequence motif – the ACGT core sequence – is functionally important in a variety of promoters that respond to different stimuli like light [12], anaerobiosis [13], jasmonic acid [14] and hormones such as salicylic acid [15], abscisic acid [16-18] and auxin [19]. This core element is present at different relative positions in multiple copies upstream of the transcription start site [20], and any alterations in this core sequence reduce the overall promoter activity significantly, for it contributes synergistically to gene expression by stabilizing the transcription complex formed on the minimal promoter [21]. Co-occurring ACGT elements are over-represented in Arabidopsis and rice genomes, emphasizing their functional relevance when compared to single ACGT core elements [22]. As discussed, the inter motif distance between these co-occurring ACGT sequence is of particular importance as promoter activation by ACGT is differentially regulated by the spacing between two copies of the motif [23]. Additionally, the copy number of ACGT elements in a promoter and distance from the transcription start site also drastically alter gene expression [24]. While most reports on the ACGT core sequence are based on *Arabidopsis thaliana*, the ACGT family of promoters (ACEs) have also been identified in wheat [25] rice [26-28] and barley [29], suggesting that ACEs are conserved across plant species.

Given that the ACGT core sequence is dispersed across promoters of various plant species; and that they occur in multiple copies with a variable number of base pairs separating them, we have attempted to analyze patterns in occurrence of ACGT core element repeats in plant genomes. We performed an *in-silico* search for ACGT elements separated by spacers of varying lengths in all identified promoters for two monocots – Sorghum and Rice, and two dicots – Arabidopsis and Soybean. Our data indicated similarities in the frequency patterns across the four plant species, with correlations for particular spacer lengths between ACGT core elements. In order to analyze if a specific sequence motif is preferred as a spacer between multiple ACGT elements across all promoters, we developed consensus sequences from all spacers observed. Additionally, we studied the evolution of co-occurring ACGT elements by analyzing their prevalence among orthologous genes in Arabidopsis, Rice and Sorghum. Further, to understand the functional significance of these elements, we used microarray data to analyze which conditions might be responsible for regulation of genes consisting of multiple ACGT *cis*-elements.

## Methods

### Data extraction

Focusing our analysis on promoters, we extracted 1 kb sequences upstream of all identified chromosomal genes from the following genomes - *Arabidopsis thaliana* (The Arabidopsis Genome Initiative v. 10, 2011), *Oryza sativa* (Rice) (International Rice Genome Sequencing Project, Build 4.0, 2009), *Glycine max* (Soybean) (US DOE Joint Genome Institute (JGI-PGF), v. 1.0, 2010) and *Sorghum bicolor* (Sorghum) (Sorghum Consortium, v. 1.0, 2009) using the NCBI Reference Sequence database [30-33]. Using a code (Additional file 1), we extracted gene annotation information (Gene ID/ Arabidopsis TAIR ID and ATG site) from Gene bank files and the corresponding 1 kb upstream region from the FASTA sequence. We searched for co-occurring ACGT elements of the form ACGT (N) ACGT, where 0 ≤ N ≤ 30 in all extracted 1 kb regions for our analysis. As it has been previously seen that cooperatively binding transcription factors are usually spaced within 25 bp, we limited our analysis to a spacer distance of 30 bp. The sequence of each spacer (region between two ACGT core elements) was extracted and the total number of occurrences for each spacer length was determined for each species. In order to test the significance of these frequencies, we used four palindromic – TAGC, CGTA, GCTA, ATGC, and four non-palindromic – AGCT, TGCA, CTAG, GATC sequences as controls. Using the PLACE database, we ensured that each of these 4 bp sequences are not conserved *cis* element themselves [34]. By performing a similar analysis on each control sequence, we compared the frequency of the ACGT (N) ACGT motif with the corresponding frequencies of control sequences for the same N.

### Spacer sequence analysis

Spacer sequences occurring in all promoters of *Arabidopsis thaliana* were analyzed to identify preferences for a particular nucleotide at each position within the spacer sequence. The percentage of A, G, C and T at each position for all spacer lengths (N = 0–30) was calculated to identify preferences at particular positions within the spacers. Since the genome wide GC content for *Arabidopsis thaliana* is known to be around 36% [35], we chose threshold occurrence percentage of 25% for C/G and 40% for A/T for a particular position in all spacers of the same length (N). Single letter IUPAC DNA codes were assigned to each position to generate consensus sequences for all spacer lengths.

### Ortholog analysis

In order to understand the mechanism of evolution of the ACGT (N) ACGT *cis*-element in the aforementioned plant species, we analyzed its predominance for N = 0–30 in all identified in-paralogs/orthologs among Arabidopsis, Rice and Sorghum [36]. Gene names for Rice were converted from MSU annotation to RAP annotation prior to analysis [37]. Frequencies for co-occurring ACGT elements were analyzed from extracted genes.

### Functional analysis

Using published microarray data from the EBI Gene Expression Atlas [38] for *Arabidopsis thaliana,* we analyzed whether genes containing multiple core ACGT *cis*-elements were unregulated/down regulated during developmental stages (embryo, seedling), by hormones (ABA, auxin, ethylene, gibberellin, jasmonic acid, salicylic acid), in different plant parts (cambium, flower, leaf, root, pollen, seed, sperm cell, stem, vegetative apex, apical root meristem) and by environmental conditions (at baseline growth temperature, disease, drought, low water potential, at optimum photosynthetic temperature, salt, 20% inhibition from optimum photosynthetic temperature, 30% inhibition from optimum photosynthetic temperature). In addition, we extended our functional analysis to Rice by extracting genes up regulated by salt and drought stress in 7 day old seedling from publically available microarray data [39]. By comparing genes regulated by a condition with genes containing multiple ACGT elements, we calculated the likelihood of occurrence by the following formula:

$$\mathrm{Likelihood}\;\mathrm{of}\;\mathrm{occurrence}=X/Y\;\mathrm{for}\;a\;\mathrm{particular}\;\mathrm{spacer}\;\mathrm{length}\;\left(N\right)$$

where X = (A ∩ B)/ B and Y = *P*(A);

A = event that a given gene is regulated (up/down) by a particular condition B = event that a given gene contains multiple ACGT elements separated by N base pairs. Further, we calculated the overall likelihood of occurrence for each condition (for N = 0–30). All conditions with likelihood of occurrence > 1.30 were subjected to further statistical analysis using the 8 control sequences described earlier.

### Statistical analysis

Wherever possible, statistical analysis was performed to determine the significance of results. The frequency of ACGT (N) ACGT was assessed for significant peaks by box and whiskers plots, with 10% and 90% whiskers. The outliers were considered as potential peaks, especially if they were present across all species. The degree of correlation between the two monocot and dicot species was calculated by taking the frequencies for N consecutive spacer lengths at a time, beginning from 0 till 30 for each of the species, where N > =6. By assuming Gaussian distribution, the Pearson’s correlation coefficient was calculated for each of the cases to determine significance. Consecutive spacer lengths of N with the highest degrees of correlation were interpreted as the most conserved spacer distances.

To identify conditions regulating ACGT (N) ACGT containing promoters over the 8 control elements, a Grubb’s outlier’s test was performed on the likelihood of occurrences for each condition, assuming the data set was normally distributed. If the ACGT (N) ACGT likelihood emerged significantly higher than the controls by this test for a certain condition, it was interpreted to be specifically regulated by that condition.

## Results

### Spacer frequency comparison shows common peaks and dips for different spacer lengths across species

**Figure 1:**
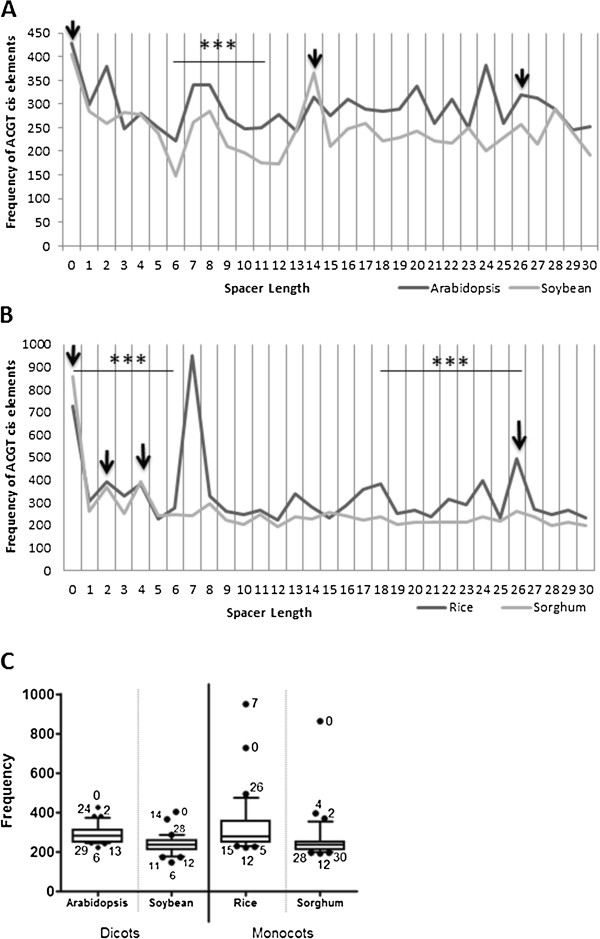
**Variation in spacer length frequencies across species.** (**A**) A comparison of ACGT elements separated by increasing spacer distance (<31) between two dicots – Arabidopsis and Soybean. Possible common peaks include 0, 14 and 26. Spacer lengths 6–11 show a high degree of correlation between the two genomes. (**B**) A similar comparison between two monocots – Rice and Sorghum, with possible peaks at 0, 2, 4 and 26. Spacer lengths 0–6 and 18–26 show significant correlation. (**C**) A 10–90% box and whiskers plot suggests 0 as the only common peak across all species. Interestingly, spacer distance 6 emerges as a common dip in dicots, whereas 12 exists as a common dip in monocots. Arrow – potential common peaks. *** *p* < 0.001.

We extracted 1 kb regions upstream of the ATG site for 33,323 genes in Arabidopsis, 49,841 genes in soybean, 30,294 genes in rice and 32,886 genes in sorghum. In total, the number of *cis*-elements of the form ACGT (N) ACGT (N ≤ 30) per 1000 promoters was found to be 271 for Arabidopsis, 341 for Rice, 246 for Sorghum and 151 for Soybean. Our data indicated an overall frequency variation for N = 0–30 for the four species under analysis. Attempting to identify conservation between monocots and dicots, we identified potential peaks at spacer lengths of 0, 14 and 26 for dicots (Figure 1A) and lengths of 0, 2, 4 and 26 (Figure 1B) across monocots. Following this, the box and whisker’s plot (10%–90%) interestingly indicated that while spacer length 0, i.e., two ACGT’s in tandem, appeared to be significantly high across all species, there was no common peak across all four genomes (Figure 1C). Frequencies for N = (0, 2, 24) for Arabidopsis, (0, 14, 28) for Soybean, (0, 7, 26) for Rice and (0, 2, 4) for Sorghum emerged to lie outside the 90th percentile. A particularly surprising observation was that spacer length 6 and the region from 11–13 had the lowest frequencies across both dicots, whereas spacer length 12 was present as a common dip between monocots.

### Local regions of conservation in monocots and dicots

Based on trends in variation of frequencies in the four species, we observed patterns of high correlation specific to monocots and dicots (Figure 1A, B). The two dicotyledonous plants involved in our study - Arabidopsis and Soybean, depicted high correlation in frequencies for spacer distance (N) = 6–11(r = 0.974; t = 8.599; *p* = 0.0010; N = 6) (Figure 1A). Similarly, Rice and Sorghum, both monocotyledonous plants, showed regions of high correlation for spacer distance (N) = 0–6 (r = 0.984; t = 12.42; *p* = .0001; N = 7) and (N) = 18–26 (r = 0.934; t = 6.921; *p* =0.0002; N = 9). Comparisons across monocots and dicots, for the most part, were found to be not as significantly correlated (r < 0.8).

### Limited preference for ACGT elements compared to random elements

**Figure 2:**
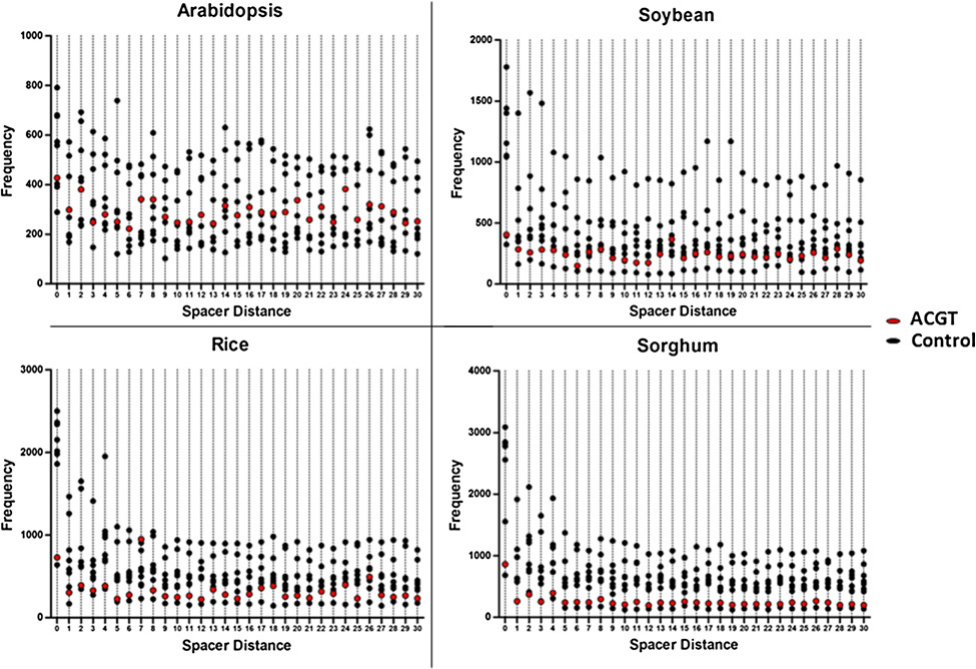
**Frequency of co-occurring ACGT elements compared to random 4 bp palindromic and non-palindromic nucleotides.** By comparing the spacer length wise frequency across all four species with 8 other random control sequences, it became apparent that co-occurring ACGT elements are not specifically preferred in terms of frequency over other random base pair sequences. Although a few high frequencies were present in rice and Arabidopsis, co-occurring ACGT elements were not predominant throughout the genomes of sorghum and soybean.

The frequency of occurrence of random 4 bp nucleotide sequences separated by varying spacer lengths in Arabidopsis, Soybean, Rice and Sorghum was determined, and compared within each species. A dot plot indicated that contrary to our hypothesis, ACGT elements are not drastically preferred over other elements across all species (Figure 2). In fact, ACGT elements usually lay around the mean of the frequencies of the control elements, with the exception of sorghum, for which the frequency of ACGT elements was lower. For Arabidopsis and Rice, spacer lengths 7 and 24 were present in higher frequencies, being surpassed only by ATGC, GATC and TGCA, which were predominantly high across all species (details in Additional file 2).

### Patterns of ACGT *cis* elements are highly conserved among orthologous gene groups

**Figure 3:**
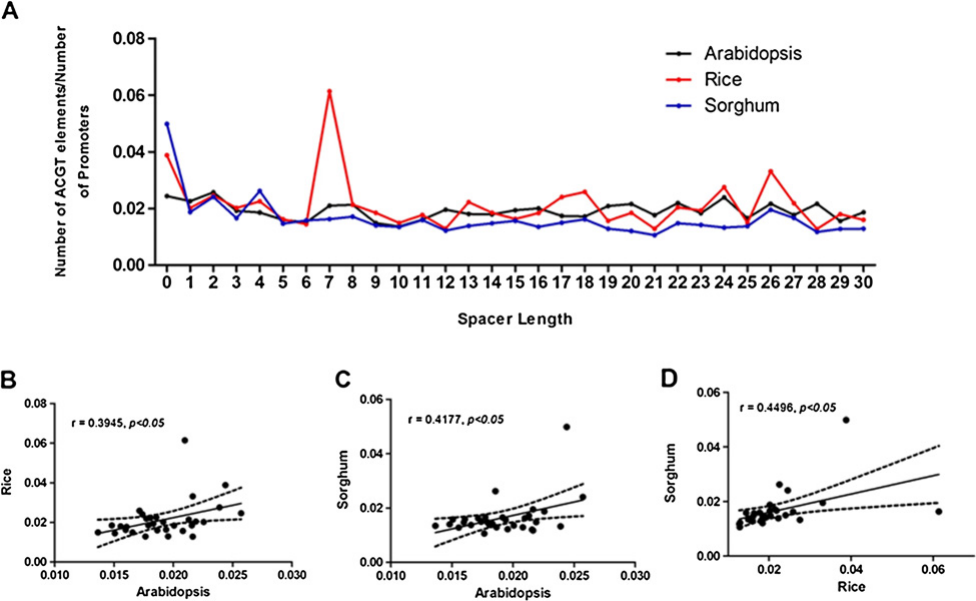
**Patterns of co-occurring ACGT elements in orthologous genes.** (**A**) Analysis of co-occurring ACGT elements in orthologous genes among Arabidopsis, Rice and Sorghum indicates similar patterns in frequency of ACGT elements per gene. Patterns were found to be significantly correlated between Rice – Arabidopsis (**B**), Sorghum – Arabidopsis (**C**) and Sorghum – Rice (**D**).

We performed a frequency analysis for ACGT repeats in promoters of all orthologous gene groups reported between Arabidopsis (12013 genes), Rice (11453 genes) and Sorghum (10575 genes). Interestingly, orthologous genes in all three species exhibit similar trends in spacer distance frequency (Figure 3A), with significant correlations across all spacer lengths for Arabidopsis and Rice (r = 0.3945, p < 0.05, n = 31) (Figure 3B), Arabidopsis and Sorghum (r = 0.4177, p < 0.05, n = 31) (Figure 3C), and Rice and Sorghum (r = 0.4495, p < 0.5, n = 31) (Figure 3D).

### The first and last positions within a spacer sequence are conserved for all spacer lengths in Arabidopsis

**Figure 4:**
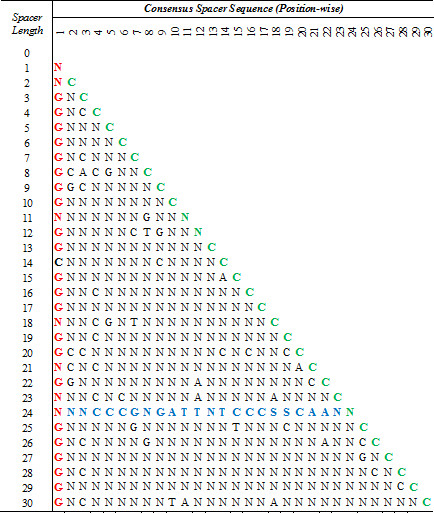
**Consensus spacer sequences of varying length with ACGT elements flanking them.** The first nucleotide for each spacer shows a clear preference for G (red), whereas the terminal nucleotide shows the conservation of C (green). Spacer length 24 is observed to be conserved to a greater extent, as compared to other spacer lengths (blue). *A* Adenine; *T* Thymine; *G* Guanine; *C*, Cytosine; *S* Guanine/Cytosine; *N* None of the bases met threshold requirements (A/T/G/C).

Consensus spacer sequences for spacers of length 0–30 were determined to be majorly composed of random nucleotides, shown as N in the sequence (Figure 4). However, the consensus sequences indicate a marked preference for G at the first position in the spacer sequences (shown in red). Similarly, a majority of the sequences indicate the presence of a C (shown in green) at the terminal end of the sequence. As an exception from all the other spacer sequences, the consensus sequence of spacer length 24 (shown in blue) is composed of conserved nucleotides at most (17/24) of the positions. Such a trend is not noted in any other spacer length, indicating a high degree of conservation for the sequence of this particular spacer length.

### ACGT repeat elements across species are preferentially regulated by salt and drought stress conditions

**Figure 5:**
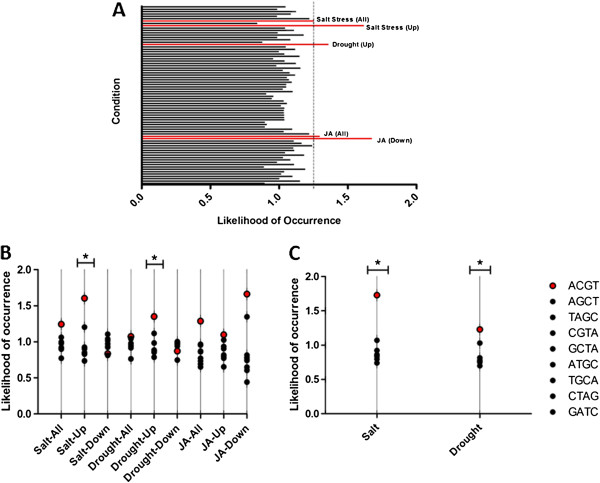
**ACGT containing promoters and their regulation under specific conditions.** (**A**) Using microarray based expression data in Arabidopsis, an analysis of 26 different conditions revealed a potential regulation of ACGT (N) ACGT containing promoters by environment conditions of salt and drought, and jasmonic acid. (**B**) Dot plots of ACGT and control elements in Arabidopsis for each of the conditions show high likelihood values for ACGT, which are significant for up-regulation by salt and drought stress. (**C**) Dot plots of ACGT and control elements for up regulation by salt and drought stress in Rice show high likelihood values for ACGT similar to those seen for Arabidopsis. * *p* < 0.5.

We identified the genes regulated by specific conditions, which also contain an upstream ACGT (N) ACGT element. The overall likelihood of occurrence was calculated for each condition, with a likelihood of 1 being that of random chance. Therefore, conditions greater than 1.3 were selected for further analysis (Figure 5A); including environment conditions such as salt and drought, and hormones such as jasmonic acid. The likelihood for each of these conditions was compared with that of the same condition for the previously described 8 control sequences. Dot plots for each of the conditions and a Grubbs’ test for outliers indicated a significant effect for ACGT containing promoters to be up-regulated by salt (Mean = 0.9791; Z = 2.21: *p* < 0.5; n = 9) and drought (Mean = 0.9539; Z = 2.21: *p* < 0.5; n = 9) (Figure 5B). An individual spacer distance -wise split up revealed considerable fluctuation in the likelihoods for each spacer length, and despite no clear pattern emerging over all functions (A 3), suggested potential spacer length specific gene regulation. Further, from microarray data for *Oryza sativa* we performed an identical analysis for our two candidate functions – salt and drought up-regulation. Both conditions showed likelihood of occurrences greater than 1.20, and the Grubbs’ test for outliers emerged to be significant for both salt (Mean = 0.9611; Z = 2.21: *p* < 0.5; n = 9) and drought(Mean =0.8453; Z = 2.21; *p* < 0.5; n = 9) up-regulation (Figure 5C). Interestingly, similar fluctuating spacer length-wise patterns were observed for this dataset.

## Discussion

The ACGT core motif forms an important class of *cis*-elements implicated in a variety of functions. Multiple ACGT motifs have been shown to form enhancer elements which bind synergistically to transcription factors for gene regulation [40]. Our data indicates that certain spacer lengths are preferred over others in plant promoters. It is possible that these spacer lengths are present in abundance due to extra stability conferred by helical phasing at these particular lengths [41]. A major finding was the peak at spacer distance (N) = 7 for rice, which is noteworthy as it is almost twice of the next highest frequency observed in the distribution. To the best of our knowledge, there are no previous reports describing an interaction between *cis*-elements in rice with such spatial constraints. Therefore, a further investigation for this observation could be interesting. Unfortunately, the peaks observed in frequency distributions were not consistent throughout the four plant species, or even among individual classes. However, a consistency in the frequency dips observed among monocots and dicots suggests a class specific mode of gene regulation. A possible explanation for the consistent dips could be that certain spacer lengths might cause sterically incompatible binding of transcription factors, explaining why they could be less preferred as compared to an otherwise compatible binding caused by other spacer lengths [42]. The theory of a class specific mode of regulation is also supported by differences in regions of high correlation in monocots and dicots.

The correlations in the patterns of ACGT repeat frequencies led us to speculate the precise mechanism of conservation of these repeats. The similar trends and subsequent functional significance of ACGT elements suggest two possible mechanisms of progression - either parallel or convergent evolution. We expect that a significant correlation in the spacer patterns of orthologous/paralogous genes groups would confirm the evolution of ACGT elements being a result of parallel evolution, whereas no correlation would suggest convergent mechanisms. Our results strongly suggest proof for parallel evolution, as patterns in ACGT elements appear to have evolved from a common ancestral gene and subsequently persisted in the descendent genes across species.

The subsequent comparison of co-occurring ACGT elements with random 4 bp nucleotides indicated that ACGT elements are not as predominant in plant promoters as compared to other random elements. This observation indicates that although a higher frequency results from a preferred occurrence, it might not be a result of conservation in the genome. Nevertheless, on analyzing the same random elements for functional preference using microarray data, ACGT (N) ACGT elements were found to be predominant in spite of lower frequencies than random elements in all promoters. This fact underscores the functional relevance of ACGT (N) ACGT *cis*-elements.

Based on our functional analysis, we deduced that co-occurring ACGT elements are involved in gene regulation in response to stress conditions in both Arabidopsis and Rice, suggesting species-wide functional significance. Genes which were up-regulated by salt and draught stress were much more likely to contain *cis*-elements of the form ACGT (N) ACGT in their promoters. This observation is supported by previous reports that multiple basic leucine zipper transcription factors, which recognize the ACGT core site, have been implicated in response under drought and high salinity conditions in Arabidopsis [16,43]. Similarly, bzip transcription factors have been shown to be involved in regulation under drought stress in Rice and Soybean [44,45]. Further, although regulation by jasmonic acid could not satisfy the criteria for the Grubs’ outlier tests, the likelihood of occurrence of genes regulated by jasmonic acid was higher than the cutoff of 1.30. With jasmonic acid’s conventional involvement in mediating stress responses in plants [46], this observation is extremely interesting in light of our findings.

Spacer sequence results showed a clear preference for G at the first and C at the terminal position for almost all spacer lengths. This validates previous reports which state that the sequence requirement of the ACGT-containing ABRE is ACGT-G G/T C [47]. The CACGTG motif, or the G-box, is recognized in rice (Kumar A, 2009), and our results clearly show a predominance of G at +2 and C at -2 positions. This result also corresponds to reports which state that the bZip class of TFs show enhanced binding to ACGT elements with the presence of the G box [48]. It can therefore be inferred, that a majority of TFs which bind to ACGT elements have stronger interactions if the flanking nucleotides are C and G. While the overall spacer sequence did not show any clear consensus sequence, spacer distance 24 showed a large amount of conservation. Spacer sequence between two ACGT motifs in a *cis*-element can be crucial for gene regulation [49]. From our frequency analysis, we determined that spacer length 24 also appears to be preferred in Arabidopsis. In light of these observations, it is possible that a spacer of 24 bases might be functionally relevant in Arabidopsis.

Determining the optimum spacer length and preferred spacer sequence could dramatically enhance promoter designing techniques. If a particular spacer length is confirmed to be implicated in regulation of a particular function, e.g. - stress response, a *cis*-element containing the ACGT repeated motif can be incorporated within promoters to give rise to sturdier and more resistant genetically modified crops [50]. Therefore, identifying the mechanism and implications of the conservation of specific spacer lengths and sequences is of prime importance for various genetic engineering techniques. Having identified spacer lengths between ACGT elements which can up-regulate gene expression in conditions of draught and salt stress, these results suggest improved methods for promoter design and creating hardy plant varieties.

## Conclusions

This is the first study which has attempted to analyze patterns of ACGT repeat *cis*-elements in four plant genomes. We established that each species exhibits preferences for particular spacer lengths and demonstrated the existence of spacer lengths preferentially avoided in monocots and dicots. This suggests that a class specific mechanism of gene regulation might be present for ACGT (N) ACGT elements. We further identified parallel evolution to be the underlying mechanism for ACGT co-occurrences across species. Moreover, by indicating that genes up-regulated by salt and drought stress are more likely to contain ACGT repeat *cis*-elements in their promoters, our *in-silico* results suggest a significant role of these elements in these pathways.

## Competing interest

Authors are not having any competing interest.

## Authors’ contributions

RM conceived the study and participated in its co-ordination. RM also gave critical inputs on designing of experiments. SS and IZ designed the experiments, conducted the analysis and drafted the manuscript. PB and SM provided valuable inputs in shaping the manuscript to its final form. All authors read and approved the final manuscript.

## Supplementary Material

Additional file 1: Table S2 Frequency analysis of control cis-elements across four plant species. A comparison of the frequency of occurrence of each of the 4 palindromic and 4 non-palindromic control elements used for N=(0-30) in -1000 bp regions of Arabidopsis, Soybean, Sorghum and Rice. Click here for file

Additional file 2: Table S3 Functional analysis of Arabidopsis and Rice genes containing ACGT *cis*-elements. The (A) total number of ACGT(N)ACGT (0<=N<=30) containing promoters in Arabidopsis that are also regulated by certain conditions, with a spacer-length wise frequency analysis, (B) likelihood of occurrence to contain ACGT(N)ACGT, calculated for each of these conditions, (C) identical analysis conducted for each of the eight control elements used previously (Additional file 1), (D) comparison of the likelihoods for ACGT and each of the controls, with conditions highlighted in green being those for which the likelihood of ACGT is significant in the Grubbs’ test for outliers (E) total number of ACGT(N)ACGT (0<=N<=30) containing promoters in Rice that are up-regulated by salt and drought stress, with a spacer length wise analysis and a similar analysis for control elements and (F) comparison of the likelihoods for ACGT and for each of the controls in Rice with both conditions emerging significant in Grubbs’ test for outliers. Click here for file
